# The impact of seasonality on the dynamics and control of *Ascaris lumbricoides* infections

**DOI:** 10.1016/j.jtbi.2018.05.025

**Published:** 2018-09-14

**Authors:** A.J. Cooper, T. Déirdre Hollingsworth

**Affiliations:** a Zeeman Institute for Systems Biology and Infectious Disease Epidemiology Research, University of Warwick, Coventry CV4 7AL, UK; b Big Data Institute, Li Ka Shing Centre for Health Information and Discovery, University of Oxford, Oxford OX1 2JD, UK

**Keywords:** Neglected tropical disease, Soil-transmitted helminth, Mass drug administration, Climatic forcing, Local elimination

## Abstract

Intestinal nematode infections affect a huge proportion of the world’s population. Increasingly these infections, particularly amongst the poorest communities, are controlled through mass drug treatment programs. Seasonal variations of climate and behaviour in these regions can be significant, but their impact on the dynamics of infection and implications for the effectiveness of any mass drug treatment program (a pulsed reduction in worm burden in hosts) is not clearly understood. Here the effect of seasonality on the dynamics of the soil-based helminth, *Ascaris lumbricoides*, is investigated using a reformulated version of the Anderson–May model for macro-parasitic infections. Explicit analytical expressions are obtained for the stable oscillatory solution over the annual cycle, which provides a means of relating times of peak numbers of eggs, larvae and mature worms to seasonal variations. Numerical and analytical techniques are then used to consider the impact of seasonality on the optimal timing of drug treatment.

Our results show that there is a relatively large window for the timing of optimal treatment, and the impact of repeated annual mass drug treatments can be substantially improved if they are timed to coincide with the months when the number of eggs and larvae are at their lowest - minimising reinfection. In terms of a more measurable quantity, in our example this corresponds to the months when the seasonal temperature is highest. Multiple annual treatments at (or close to) the optimal time each year are predicted to achieve local elimination in the community, whereas treatment at other times has a more limited impact. A key finding is that even for pronounced seasonality, perturbations in mean worm burden, and hence seasonal variation in observed egg output, may be small, potentially explaining why seasonal effects have been overlooked. Taken together these results suggest that seasonality of soil-transmitted helminths requires further experimental, field and mathematical study if the impact for mass drug administration programs is to be exploited.

## Highlights

• Model developed to assess impact of seasonality on *Ascaris lumbricoides* infections.
• Analytical expressions relate egg and worm numbers to seasonal variations.
• Reduction in seasonal perturbation through the life cycle.
• Utilising seasonality for optimal timing of annual treatment has profound benefits.
• Exploit seasonality to reduce number of treatment rounds for local elimination.

## 1 Introduction

Intestinal nematode infections affect up to 1/3 of the world’s population (approximately 1.4 billion people worldwide), with *Ascaris lumbricoides* - the intestinal roundworm - the most common (Pullan et al., 2014). Most cases are asymptomatic, but infection can result in pulmonary and sometimes severe gastrointestinal complaints. *Ascaris* infection is most common in areas of poor sanitation and can lead to malnutrition, vitamin and mineral deficiencies and impair growth and cognitive function (Bethony et al., 2006).

Mass drug treatment programs as a means of controlling *Ascaris* infection, and other neglected tropical diseases, are widely used and have increased in recent years. A single treatment cannot be effective in controlling the infection since it acts only on worms in the host, which leaves a reservoir of eggs and larvae in the environment allowing the life cycle to continue. Therefore, since the drugs are relatively inexpensive and often donated (www.who.int/neglected_diseases/en), entire communities at risk are often offered multiple rounds of treatment. However, the optimal strategy for delivering the most effective community-based treatments is still open to question (Anderson, Hollingsworth, Truscott, Brooker, 2012, Anderson, Turner, Truscott, Hollingsworth, Brooker, 2015). Seasonal treatment has been evaluated for Schistosomiasis (Augusto et al., 2009), where the effect of treatment was found to be enhanced if administered during the low transmission season. Uncertainties in treatment timing, treatment intervals and for how long to continue with treatment with regard to *Ascaris* infection remain. Understanding the impact of seasonality on the dynamics of infection may help to address some of these issues.

Transmission of *Ascaris* infection between hosts is achieved by ingestion of infective larvae originating from eggs passed into the external environment in faeces. This route of transmission is very successful in poor communities in developing countries where there is limited sanitation available. Eggs passed in faeces are not immediately infective, but must undergo further development in the environment before they become infective. This development stage is thus subject to environmental factors such as temperature, moisture levels and exposure to sunlight, which can be highly variable throughout an annual cycle.

Seasonal influence on *Ascaris* infection has been observed in several villages in Sri Lanka (Gunawardena et al., 2004), where correlations were found between numbers of wet days and *Ascaris* infection and re-infection levels. Other studies have found seasonal trends in prevalence and reinfection rates, both in relation to temperature variations and rainy seasons (Gungoren, Latipov, Regallet, Musabaev, 2007, Pan, Ritchie, Hunter, 1954, Seo, Cho, Choi, 1979). Changes in environmental conditions are also known to affect the development characteristics of *Ascaris* eggs and larvae. Experimental investigations have shown that egg and larvae development and survival characteristics are influenced by temperature (Arene, 1986, Kim, Pyo, Hwang, Park, Hwang, Chai, Shin, 2012, Wagner, Polley, 1999). For example low temperatures were found to delay egg development, but lead to enhanced survival, whereas higher temperatures increase the rate of egg development but survival chances are diminished. Seasonal differences in rates of embryonation of *Ascaris suum* eggs in the outdoor environment have also been observed (Larsen and Roepstorff, 1999), suggesting that temperature dependency may have important epidemiological consequences. Therefore temperature (or other environmental or behavioural) variation over a year is likely to lead to different infection dynamics throughout the season. This suggests that the impact of mass drug treatment programs could vary significantly depending on when the drugs are administered, and that seasonality is an important factor to consider in the design of community-based treatment programs.

The purpose of this paper is to use mathematical models to establish whether seasonal effects are an important consideration in the dynamics and control of *Ascaris* infection. Mathematical models are a useful tool for assessing the impact, and developing the understanding, of the role of seasonality in the transmission of *Ascaris* infection. Such models can allow for different aspects of the transmission process to be isolated, and often lead to suggestions for paths of further experimental and field investigation.

In this paper a mathematical model developed from the Anderson and May (1991) model for macro-parasitic infections is used, together with an assumed generic seasonal temperature distribution, and egg and larvae development characteristics inferred from the previous experimental investigation of Arene (1986). Mathematical techniques are used to determine analytical solutions for the stable, but seasonally varying, states of the four stages of the life cycle of *Ascaris lumbricoides*. This is a useful tool which allows the peaks in egg numbers or mean worm burden to be related to the underlying seasonal temperature profile. The influence of various parameters associated with the transmission process, such as egg development rate, egg survival and larvae death rate, on the infection dynamics is investigated. The impact of seasonal variation on the dynamics of infection following drug treatment is then addressed. The effectiveness of the drug treatment in the model is taken to be a combination of the drug efficacy and the proportion of people who are treated, and so implicitly incorporates any effects of non-random compliance. The aim is to establish whether seasonal effects could be exploited in order to maximise any effects of multiple drug treatment strategies and determine areas for further research.

## 2 Mathematical model

The life cycle of *Ascaris lumbricoides* can be described as follows. Fertile eggs in the environment begin to develop and become infective after about 10–30 days depending on environmental conditions, which defines the time, *τ_E_*, for maturation of eggs to infective larvae. Infective eggs are ingested by humans at a rate *β*, after which the larvae hatch and make their way from the intestinal system into the bloodstream and on to the lungs. The larvae mature further in the lungs, then ascend the bronchial tree to the throat and are swallowed. Upon reaching the small intestine they develop into adult worms. The process of ingestion of infective eggs to egg-laying adult worm takes about 50–80 days (which defines the maturation time *τ_J_*). The lifetime of the adult worms is about 1–2 years. The life cycle is completed when eggs are excreted back into the environment. During this life cycle only a proportion of eggs, *s_E_*, will survive to become infective. Similarly only a proportion, *s_J_*, of juvenile worms survive to maturity. Infective larvae can survive in the environment for about 24–84 days, which introduces a larval death rate *μ_L_*. Loss of mature worms can be effected either through death of the mature worm (death rate *μ_M_*) or loss of the host (death rate *μ_H_*).

The Anderson and May (1991) model describing the process of soil-transmitted helminth infection uses two coupled differential equations for the larval population in the environment and the mature worm population in the host. This model includes time delays to account for the maturation time of eggs to infective larvae, and time from infection to development of worms capable of producing eggs. Since seasonal changes will affect egg numbers, and the viability of eggs and larvae, this model is rewritten as four coupled equations for the life-cycle stages: fertile, non-infective eggs (*E*) and infective larvae (*L*) in the environment, and juvenile worms (*J*) and mean number of mature worms (*M*) in the host, so that expressions for the seasonal variation of egg maturation times, etc. can be included explicitly.

The governing equations for a host community of size *N* are then as follows:

(1) $$\begin{array}{ccc} \frac{dE}{dt} & = & \sigma N\phi \left(M\right)\lambda \left(M\right)M-\frac{E}{\tau_{E}}, \end{array}$$

(2) $$\begin{array}{ccc} \frac{dL}{dt} & = & \frac{s_{E}E}{\tau_{E}}-\left(\beta N+\mu_{L}\right)L, \end{array}$$

(3) $$\begin{array}{ccc} \frac{dJ}{dt} & = & \beta L-\left(\frac{1}{\tau_{J}}+\mu_{H}\right)J, \end{array}$$

(4) $$\begin{array}{ccc} \frac{dM}{dt} & = & \frac{s_{J}J}{\tau_{J}}-\left(\mu_{M}+\mu_{H}\right)M. \end{array}$$

These equations set the timescales for maturation of eggs to infective larvae, given by *τ_E_*, and for juvenile to mature worms, given by *τ_J_*. Here *σ* is the proportion of mature female worms in the population, *ϕ* is the mating probability and *λ* is the mean rate of egg production per mature worm (fecundity).

The mean worm burden is assumed to have a negative binomial distribution, and the corresponding fecundity and mating functional forms are those used in Truscott et al. (2014). To allow for density dependent effects, where the production of eggs is constrained when there are large numbers of mature worms, the egg production term is of the form:

(5) $$\lambda \left(M\right)=\frac{\lambda_{0}z}{{\left[1+M\left(1-z\right)/k\right]}^{k+1}},$$

where *k* is the negative binomial aggregation parameter and *z* is the density dependent fecundity parameter. For polygamous worms the mating function is of the form:

(6) $$\phi \left(M\right)=1-{\left(\frac{1+M(1-z)/k}{1+M(2-z)/k}\right)}^{k+1}.$$

Note that *ϕ* saturates to unity for large values of *M*, but is less than unity when mean worm burdens are low. Other forms for *λ* and *ϕ* have been considered and corresponding results for a random distribution are included in the supplementary material.

### 2.1 Seasonal parameters

All parameters concerned with parasite stages in the environment may be subject to seasonal variation, ranging from temperature changes across the year, to the distribution of annual rainfall. Experimental evidence suggests definite links between temperature and the development time of *Ascaris lumbricoides* eggs and the subsequent egg and larvae survival characteristics. The rate of development of eggs has been shown to increase with temperature. However, at higher temperatures the eggs have less ability to hatch, and the infective larvae have shorter lifetimes (Arene, 1986, Kim, Pyo, Hwang, Park, Hwang, Chai, Shin, 2012, Wagner, Polley, 1999).

This paper is concerned with whether seasonality could play a significant role in the dynamics of *Ascaris* infection, so general trends (analytical formulae) are introduced for the seasonal parameters *τ_E_, s_E_* and *μ_L_*, based on an assumed temperature profile and the results presented in Arene (1986). However, since temperature is not included specifically in the model, inferences can be made based on the general trends in the seasonal parameters. For example warm and moist environments enhance the survival of *Ascaris* eggs. Therefore rainfall, which provides the essential moisture, leads to more viable eggs and infective larvae (Crompton and Pawlowski, 1985).

Suppose the temperature Θ has an annual cycle, with average temperature Θ*, then the following functional form can be assumed:

(7) $$\Theta =\Theta^{*}\left(1-ɛ\hat{\Theta }\cos\omega t\right),$$

where ω=2π/365 and ε is a scaling parameter corresponding to the degree of seasonality. This gives a peak in temperature in the middle of the year. Given the trends of egg development time decreasing with temperature, less chance of egg survival as temperature increases and reduced larvae lifetime at higher temperatures, the following functional forms are assumed for the seasonal parameters:

(8) $$\begin{array}{ccc} \tau_{E} & = & \tau_{E}^{*}\left(1+ɛ{\hat{\tau }}_{E}\cos\omega t\right), \end{array}$$

(9) $$\begin{array}{ccc} s_{E} & = & s_{E}^{*}\left(1+ɛ{\hat{s}}_{E}\cos\omega t\right), \end{array}$$

(10) $$\begin{array}{ccc} \mu_{L} & = & \mu_{L}^{*}\left(1-ɛ{\hat{\mu }}_{L}\cos\omega t\right), \end{array}$$

where $\tau_{E}^{*},s_{E}^{*}$ and $\mu_{L}^{*}$ are the average values for egg maturation time, proportion of eggs surviving to maturity and larvae death rate respectively, and ${\hat{\tau }}_{E},$ ${\hat{s}}_{E}$ and ${\hat{\mu }}_{L}$ (all positive) control the parameter specific level of seasonality.

Parameter values used throughout correspond to those given in Anderson and May (1991) and Fowler and Hollingsworth (2016) and are listed in Table 1.

**Table 1:** Parameter values.

Symbol	Meaning	Value
$\tau_{E}^{*}$	Average delay from release of eggs to development of larvae	20 days
*τ_J_*	Delay from infection of host to production of eggs	65 days
$1/\mu_{L}^{*}$	Average life expectancy of larvae	56 days
1/*μ_M_*	Life expectancy of mature worm	1 year
1/*μ*	Life expectancy of host	50 years
$s_{E}^{*}$	Average proportion of eggs surviving to become infective	0.7
*s_J_*	Proportion of juvenile worms surviving to maturity	0.5
*σ*	Proportion of female worms in the population	0.5
*N*	Number of hosts in the population	100
*λ*_0_	Per capita rate of egg production by female worms	2 × 10^5^ day${}^{-1}$
*k*	Negative binomial aggregation parameter	0.7
*z*	Density dependent factor for egg production	0.93

## 3 Stable solution

**Fig. 1:**
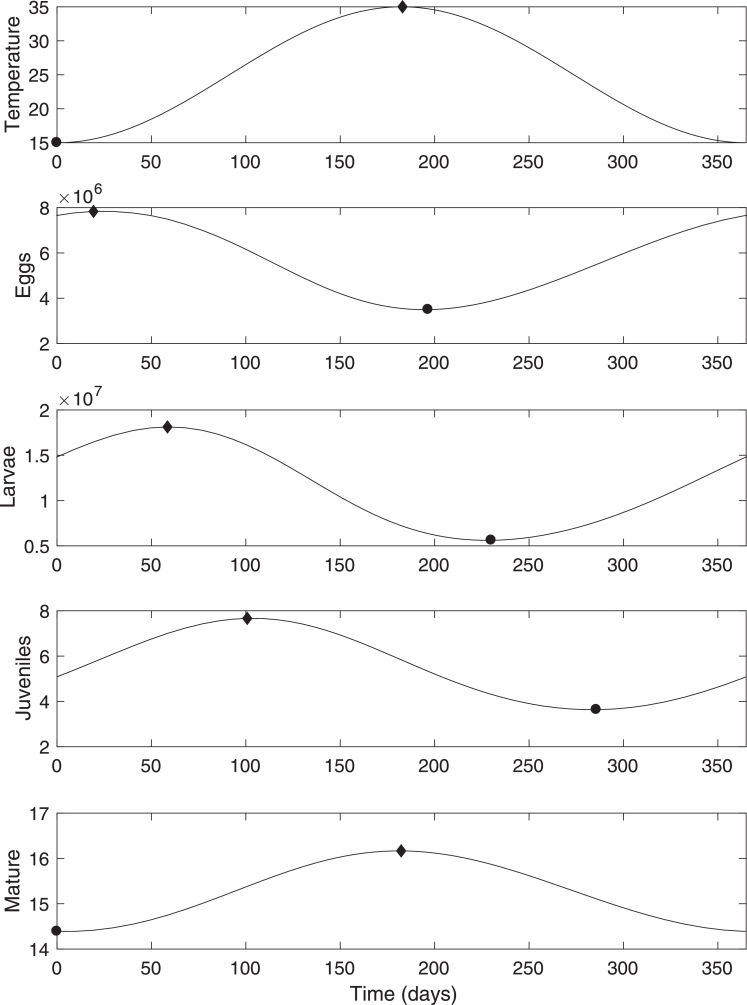
Annual stable oscillatory solution. Circles denote minimum positions and diamonds maximum positions. Here ɛ=0.4 and *β* is set by fixing the average value of *M* to be 15.

Consider the state where all four populations of the life cycle reach a stable solution. In the absence of seasonal forcing this would be the equilibrium state, where the population of each stage reaches a constant level. In the presence of weak periodic seasonal forcing the stable solution is now an oscillatory solution with the same period as the forcing. An example numerical solution is given in Fig. 1.

It can be seen from the results that the peak in egg numbers occurs a short time after the minimum in temperature. This peak then pulses through the system, revealing the delay from the peak in egg numbers through to the peak in mean mature worms. Note that the relatively large value of ε, which is a measure of the degree of seasonality, results in a relatively small variation in *M*. This apparently small effect of seasonality on the mature worm burden should not be underestimated however, since it is the seasonal effect on the whole of the life cycle which is important and will become evident later. The next section uses analytical techniques to derive explicit expressions for this stable oscillatory solution, and the pulsed behaviour through the system. The ability to relate the distribution of mean worm numbers to the distribution of egg numbers in the environment will be useful since this is likely to have implications for the timing of drug treatments, which will be addressed later.

### 3.1 Analytical approximation

The amplitude and phase of each stage of the life cycle can be determined analytically by assuming that the seasonality acts as a small perturbation to average values, and that the response is a perturbation to the equilibrium values, so that

(11) $$\begin{array}{ccc} E(t) & = & E^{*}+ɛ\hat{E}e^{i\omega t} \end{array}$$

(12) $$\begin{array}{ccc} L(t) & = & L^{*}+ɛ\hat{L}e^{i\omega t} \end{array}$$

(13) $$\begin{array}{ccc} J(t) & = & J^{*}+ɛ\hat{J}e^{i\omega t} \end{array}$$

(14) $$\begin{array}{ccc} M(t) & = & M^{*}+ɛ\hat{M}e^{i\omega t} \end{array}$$

where [E*, L*, J*, M*] are the time-invariant equilibrium values established in the absence of seasonality, $\left[\hat{E},\hat{L},\hat{J},\hat{M}\right]$ are the amplitudes resulting from seasonality, and ε is the (small) seasonality parameter. Note that taking the real part of the exponential in Eqs. (11)–(14) recovers the cos (*ωt*) form assumed for the seasonal parameters in Eqs. (8)–(10). The advantage of this formulation is that the impact of individual seasonal parameters can be determined separately, allowing the effects of each parameter to be established, with the overall effect given by the sum of the individual solutions.

Solutions are determined by substituting the forms in Eqs. (11)–(14) into the governing equations and equating powers of ε. Details appear in the Appendix. For all cases the leading-order solution determines the equilibrium solution, which is the steady solution in the absence of seasonality. This takes the form:

(15) $$\begin{array}{c} E^{*}=\sigma N\lambda^{*}\tau_{E}^{*},\;L^{*}=\frac{s_{E}^{*}}{\tau_{E}^{*}\beta_{L}}E^{*},\;J^{*}=\frac{\beta }{\xi_{J}}L^{*},\;M^{*}=\frac{s_{J}}{\tau_{J}\mu_{M}}J^{*}, \end{array}$$

where $\beta_{L}=\beta N+\mu_{L}^{*}$ and $\xi_{J}=1/\tau_{J}+\mu$.

The *O*(ε) solutions give the corrections due to seasonal forcing. For the case where only *τ_E_* is seasonal the corrections are as follows:

(16) $$\begin{array}{ccc} \hat{E} & = & \frac{{\hat{\tau }}_{E}}{\sqrt{1+\omega^{2}\tau_{E}^{*2}}}e^{-i\theta }E^{*}, \end{array}$$

(17) $$\begin{array}{ccc} \hat{L} & = & \frac{e^{-\frac{i\pi }{2}}\omega \tau_{E}^{*}\beta_{L}}{\sqrt{\omega^{2}+\beta_{L}^{2}}}\;\hat{E}e^{-i\psi }, \end{array}$$

(18) $$\begin{array}{ccc} \hat{J} & = & \frac{\xi_{J}}{\sqrt{\omega^{2}+\xi_{J}^{2}}}\;\hat{L}e^{-i\alpha }, \end{array}$$

(19) $$\begin{array}{ccc} \hat{M} & = & \frac{\mu_{M}}{\sqrt{\omega^{2}+\mu_{M}^{2}}}\;\hat{J}e^{-i\gamma }, \end{array}$$

where $\theta =\tan^{-1}\left(\omega \tau_{E}^{*}\right),$ $\psi =\tan^{-1}\left(\omega /\beta_{L}\right),$ $\alpha =\tan^{-1}\left(\omega /\xi_{J}\right)$ and $\gamma =\tan^{-1}\left(\omega /\mu_{M}\right)$ correspond to the phase changes which occur through each stage of the life cycle. It is precisely these phase shifts which produce the pulse through the system and the delay from peak in egg numbers through to the peak in mature worm levels. From these results it can be seen that $\tau_{E}^{*}$ determines both the equilibrium distribution of egg numbers, and establishes the phase difference between the egg and temperature profiles. The phase difference in the larvae is dependent on the parameters *βN* and *μ_L_* and again this is carried through to *J* and *M*. The phase of the juveniles are dependent on the maturation time *τ_J_* and that of the mature worms on the death rate of the mature worms *μ_M_*. The amplitudes of the seasonal perturbations are successively reduced through the life cycle. The successive reduction in perturbation amplitude is significant, as even if the mean worm burden appears relatively constant, there may still be strong seasonality in the life cycle, particularly in the reservoir of eggs and larvae. The importance of this in terms of treatment programs is discussed later.

If it is assumed that only one of the survival functions, *s_E_* or *μ_L_*, is seasonal, then there is no first-order seasonal correction to the egg distribution. This is a consequence of only considering the egg production term to leading order as detailed in the Appendix. The remaining stages in these cases have the same subsequent phase changes as above (*ψ, α, γ*). Detailed expressions are given in the Appendix.

**Fig. 2:**
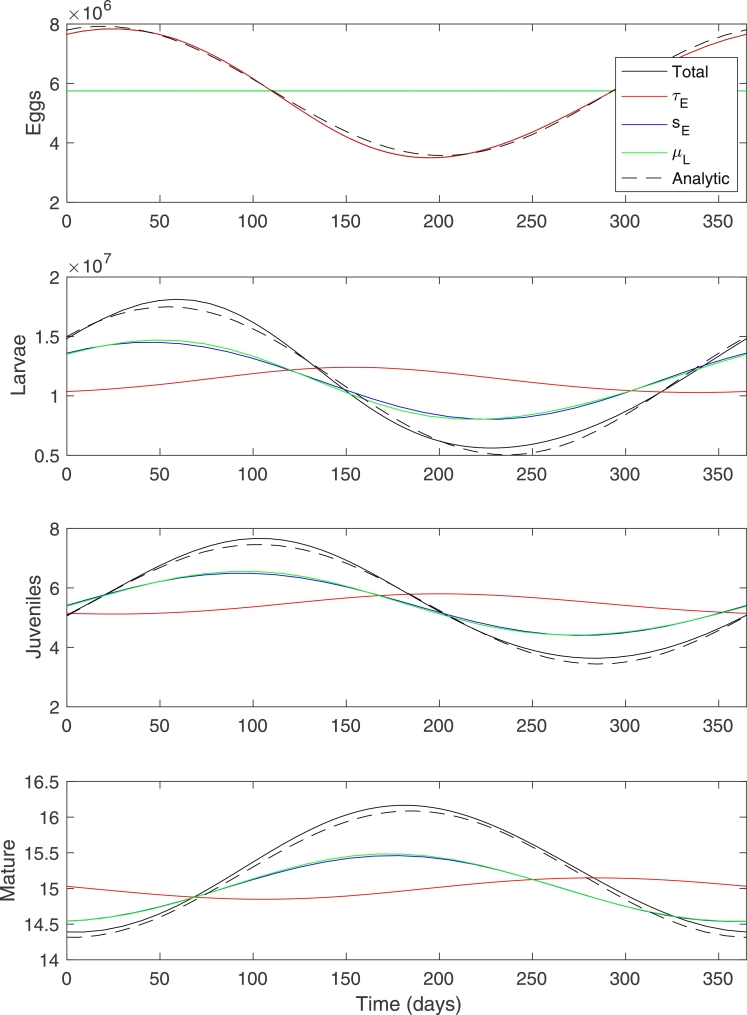
Effect of the different seasonal parameters $\tau_{E}^{*},$$s_{E}^{*}$ and $\mu_{L}^{*}$ on the steady oscillatory solution, and comparison between the total numerical and analytical solutions. (ɛ=0.4,$M^{*}=15,$${\hat{\tau }}_{E}=1,$${\hat{s}}_{E}=1,$${\hat{\mu }}_{L}=1$).

The effects of the different seasonal parameters are shown in Fig. 2, together with the combined effect compared to the analytical approximation. Good agreement is found even when ε is relatively large. As can be seen the variation in eggs is dominated by the contribution from the variation in *τ_E_* only, and shows that the leading order approximation to the egg production term is sufficient to capture the behaviour. The seasonal impact on the remaining stages is largely governed by variations in *s_E_* and *μ_L_*.

An important point to note here is that these results are for the simple sinusoidal functional forms assumed. However, any regular annual pattern can be broken down into a sum of such sinusoidal forms with different amplitudes. Each of these would generate sinusoidal perturbations in the underlying populations which would sum together to give the overall impact of any annual pattern. Therefore this analytical solution can be applied to more complicated seasonal patterns.

## 4 Treatment

Antihelmintic drugs (such as Mebendazole) are known to be highly effective against *Ascaris* infection (de Silva et al., 1997), and act on both the juvenile and mature worms in the intestine. Drug efficacy is typically in the region of 90% or more for *Ascaris lumbricoides*.

Mathematically, if it is assumed that drug treatment has effectiveness *X*, and the drug is administered at a specified treatment time *t*, then

J→(1−X)J(t),M→(1−X)M(t).

**Fig. 3:**
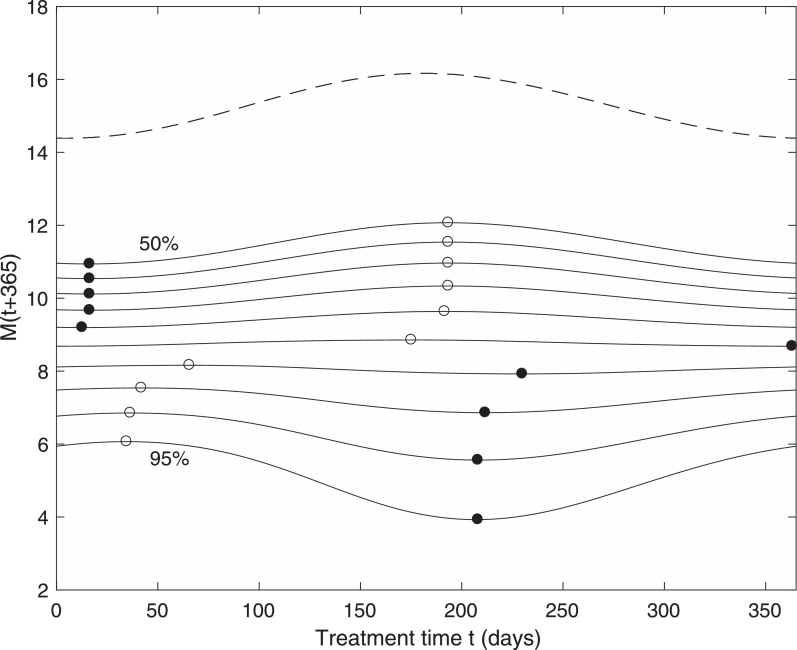
Effects of a single treatment across the annual cycle on bounce back of mature worms one year after treatment, for treatment effectiveness between 50% and 95% in 5% intervals. Filled circles denote the minimum points and open circles denote maximum points across the year. The dashed line is the pre-treatment level. (ɛ=0.4,$M^{*}=15$).

Despite considerable reductions in worm numbers following treatment, the levels of infection bounce back, sometimes relatively quickly, owing to the abundance of eggs and larvae which remain in the environment. However, as already shown, the level of eggs and larvae in the environment is subject to seasonal variation, so that the timing of treatment may be crucial in gaining the most impact. If the model is run forward a year from the time of treatment, then it is of interest to know how the mean number of mature worms in the population recovers. This is shown in Fig. 3 for treatment administered throughout the year, and for different values of treatment effectiveness.

For low drug effectiveness (<70%) the results indicate that the best time to treat is early in the year, which, referring back to Fig. 1, corresponds to when the number of mature worms is near a minimum. For highly effective treatments (>70%), as is usually the case, the best time to treat appears to be mid-way through the year, which coincides with when egg and larvae numbers are at a minimum. Note that for high effectiveness, the optimum treatment time is when the pre-treatment state is near a maximum, indicating that it is not the solely the initial seasonal distribution of mature worms which determines the degree of bounce back. Rather it is a more complex combination of seasonal factors which determines the bounce-back rate.

**Fig. 4:**
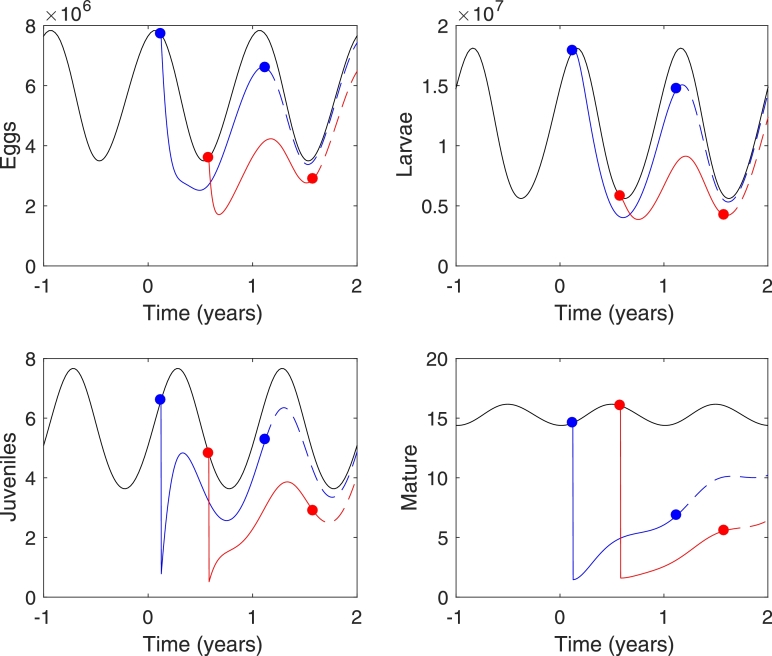
Effects of a single treatment. Time scale starts from zero in treatment year. Cases plotted include most effective (red) and least effective (blue) times to treat in terms of recovery of mature worms. Black lines indicate the untreated case. Dots indicate time of treatment and time one year after treatment. Dashed lines indicate population levels if no further treatment occurs. Treatment effectiveness is taken to be 90%. (For interpretation of the references to colour in this figure legend, the reader is referred to the web version of this article.)

Extending these ideas for highly effective treatments full numerical simulations were carried out in order to show the effects of the timing of a single treatment. Fig. 4 shows the full temporal dynamics following treatment at the most and least effective times when the bounce back of mature worms is minimised and maximised respectively. This clearly shows that the bounce back in mean worm burden is least (red line) when egg and larvae numbers at treatment time are near a minimum, even though the mean number of mature worms in this case is higher than for the least effective treatment time. It is also significant to note that a year after treatment, egg and larvae numbers are again near a minimum for the most effective treatment time. This will have implications for multiple treatment strategies.

**Fig. 5:**
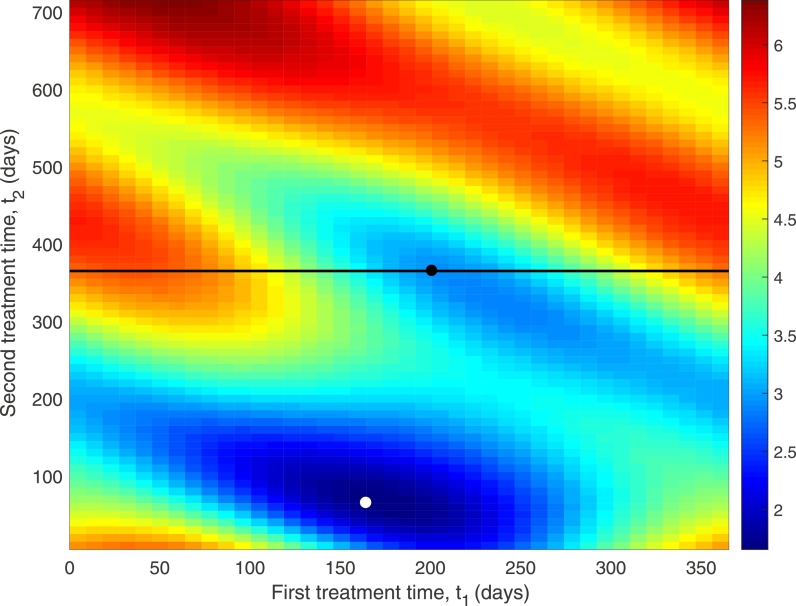
Timing of second treatment - value of *M* a year after second treatment. Treatment effectiveness = 90%, ɛ=0.4,$M^{*}=15$. White dot shows absolute minimum in *M* a year after 2 treatments. Black line shows second treatment a year after the first, with the minimum location along this line indicated by the black dot.

A single treatment is never sufficient to control the infection, so the question of when to administer a follow-up treatment is also investigated. It is assumed that the initial treatment occurs at time *t*_1_, with a subsequent follow-up treatment administered after time *t*_2_, up to two years after the initial treatment. Fig. 5 plots the value of the mean worm burden one year after the second treatment as a function of all possible initial and follow-up treatment times. Measuring the mean worm burden at one point in time is an approximation to the total burden, which is given by the integral of the burden over a specified time period (Medley et al., 1993). This approximation, however, is sufficient to demonstrate how effective the timing of the drug is in reducing the burden.

Results show that there is an absolute minimum in the bounce back after two treatments for an initial treatment at *t*_1_ ≈ 160 days, with follow-up treatment at *t*_2_ ≈ 70 days later. Referring back to Fig. 4 this most effective second treatment time appears to coincide with the minimum in eggs and larvae numbers observed soon after the initial treatment. Also shown in Fig. 5 is the minimum point for a follow-up treatment at $t_{2}=1$ year after initial treatment. This occurs at *t*_1_ ≈ 200 days into the year (close to the single treatment optimum), and is when the second minimum in egg and larvae numbers is observed in Fig. 4. Further investigation could continue with this strategy and investigate whether tailored timing of multiple treatments could lead to local eradication of the worms, and how many treatments would be required to do so. However, a more practical strategy, which is developed below, is to investigate multiple annual treatments, when during the season to administer them, and how seasonal forcing may influence the number of treatments required for local eradication.

Other evidence to support the influence of egg and larvae numbers on treatment effectiveness is the effect changing the seasonal parameters has on the most effective treatment time. Returning to the analytical expressions for the phase changes of each worm stage, Eqs. (16)–(19), it can be seen that various parameters, notably $\tau_{E}^{*},\mu_{L}^{*},\tau_{J}$ and *μ_M_*, affect the phase terms of the stable oscillatory solution and also the magnitude of each of the life-cycle stages. It is of interest to know how variations in these parameters affect the phases, and subsequently what influence this has on the bounce back in worm numbers after treatment throughout the annual cycle. It is found that changes to $\tau_{E}^{*}$ and *τ_J_* affect the time in the season at which eggs and larvae are at a minimum respectively, but do not have a significant influence on the time of the minimum bounce back in *M* (changes to $\tau_{E}^{*}$ have little effect on mature worm levels, a shorter maturation time *τ_J_* produces slightly larger bounce back). Changes to the parameter *μ_M_* have little effect on the minimum bounce back time, but does affect the degree of bounce back (a shorter mature worm lifetime implies greater bounce back). The parameter $\mu_{L}^{*},$ the death rate of the larvae, has the most influence on the dynamics, with the treatment time which minimises bounce back varying between approximately 180 and 210 days for mean larvae lifetimes of 28 and 84 days respectively. Also a shorter larvae lifetime is found to produce fewer larvae, higher mean worm numbers, but less bounce back after treatment. Overall the seasonal effects due to egg and larvae parameters are found to have the most influence on the seasonal characteristics of the response to treatment.

### 4.1 Multiple treatments

**Fig. 6:**
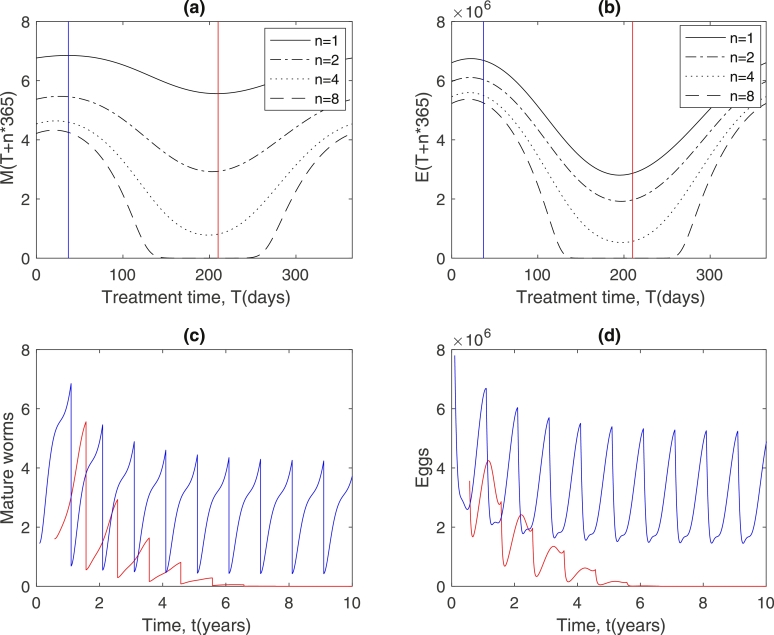
Effect of multiple annual treatments. (a) Variation in mean worm burden *n* years after initial treatment, as a function of treatment time, *T*. (b) Corresponding variation in egg numbers with treatment time. Red line indicates most effective time to treat (*T* ≈ 210 days), blue line indicates least effective treatment time (*T* ≈ 37 days). (c) Variation in mean worm burden in actual time, *t*, for most effective treatment time (red) and least effective treatment time (blue). (d) Variation in egg numbers throughout the treatment cycle at most effective (red) and least effective (blue) treatment times. (ɛ=0.4,$M^{*}=15,$ treatment effectiveness = 90%.). (For interpretation of the references to colour in this figure legend, the reader is referred to the web version of this article.)

Typical mass drug treatment programs offer a number of periodic treatments, which leads us to investigate the strategy of regular (annual) multiple treatments, and the influence of seasonal forcing in this case. Fig. 6 shows the effects of successive annual treatments applied at different times across the year. The results show that significant differences occur depending on what time of the year the treatments are applied. Fig. 6(a) shows the level of bounce back in mean worm burden one year after the initial treatment, and the corresponding levels following 2, 4, and 8 successive annual treatments. It can be seen that after each treatment round the mean worm burden falls significantly. There is evidence of local elimination, driving the mean worm population below threshold, after 8 treatments if treatment is applied around T=200 days, but the infection persists after this number of rounds of treatment if the treatment is administered near the beginning or end of the year. This effect is reinforced because the relative egg numbers also successively fall for treatment near the middle part of the year (Fig. 6(b)). This effect can be clearly observed in Fig. 6(c) and (d) which shows the levels of *M* and *E* respectively through time for the most effective time to treat (*T* ≈ 210 days) and for the least effective treatment time (*T* ≈ 37 days). It can be seen from these results that local elimination may be possible if treatment is administered in the middle of the year, but infection persists at other times. This suggests that seasonal variation needs to be carefully considered and could be exploited in order to maximise the effects of drug treatment within a community. The results presented here suggest that for the test case investigated there is approximately a 6 week window, around the annual peak in temperature, for the administration of drugs to be optimal, and that the optimum treatment time is robust in terms of the number of treatments administered. This is significant both in terms of the scope for designing effective treatment programs and the potential for successfully administering them.

A point to note is that in this test case the effect of seasonality on the variation in mean worm burden throughout the year is relatively small - see Fig. 4 where the deflection in mean worm burden from the equilibrium value is about 2 (which is unlikely to be detectable). However, despite this the results in Fig. 6 show that the seasonal influence over multiple treatments can still be very significant. This is very significant since even if the mean worm burden appears relatively constant there might be strong seasonality that could be exploited programmatically.

**Fig. 7:**
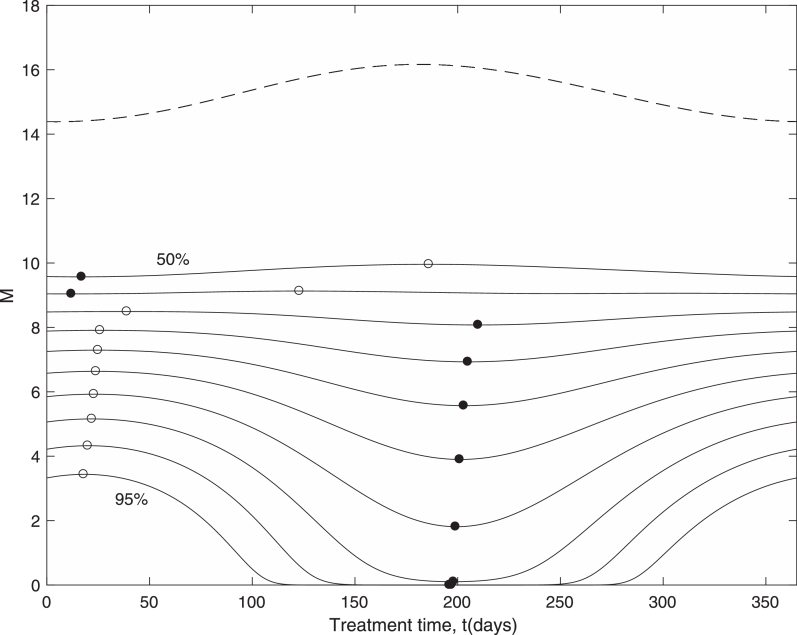
Effects of 8 annual treatments across the annual cycle on mature worms, for treatment effectiveness between 50% and 95% in 5% intervals. Filled circles denote the minimum points and open circles denote maximum points across the year. The dashed line is the pre-treatment level. (ɛ=0.4,$M^{*}=15$).

Another important consideration is the trade off between treatment timing and treatment effectiveness. In Fig. 3 it is shown that for a single treatment a higher effectiveness is generally better regardless of timing. After multiple treatments, however, it is found that less effective treatments administered over multiple years at the optimum time can achieve a better result than highly effective treatments applied at the worst time. For example Fig. 7 shows that after 8 annual treatments, treating at the optimum timing for 75% effectiveness has more impact than the worst timing for 90% effectiveness. This could be an important consideration in the design of treatment programs.

**Fig. 8:**
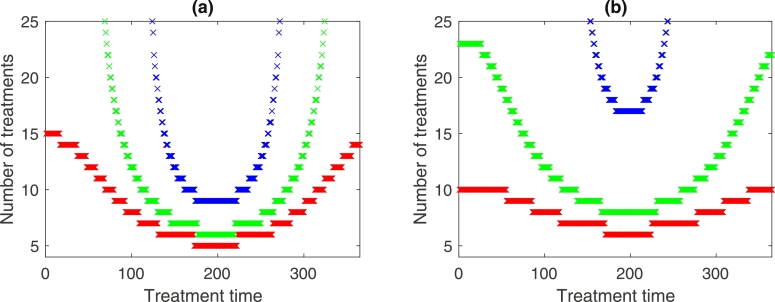
Number of treatments (up to 25) leading to local elimination for different equilibrium values of mean worm burden: red: $M^{*}=10,$ green: $M^{*}=12,$ blue: $M^{*}=15,$ with drug effectiveness = 90%. (a) ɛ=0.4, (b) ɛ=0.2. (For interpretation of the references to colour in this figure legend, the reader is referred to the web version of this article.)

So far all the results have been for a mean equilibrium worm burden of $M^{*}=15$. The impact of different values of M* on the number of treatments leading to local elimination is considered in Fig. 8 for two different levels of seasonality defined by ɛ=0.4 and ɛ=0.2. Here local elimination is assumed when the mean worm burden falls below a threshold value of 0.01, and there is no subsequent recovery in the mature worm population. Values of $M^{*}=10,12$ and 15 are used, and the results show, as would be expected, that the number of treatments leading to local elimination increases with M*. Also evident is that the minimum number of treatments required remains near the middle of the year for all worm burdens considered. For the value of $M^{*}=10,$ local elimination is suggested possible across the whole of the year, but many more treatments would be required outside the central time of the year (for example, when ɛ=0.4, local elimination is possible with 5 treatments administered in the middle of the year compared to 15 treatments in the early or late part of the year). As M* is increased the range of treatment times which leads to local elimination becomes smaller, indicating that the impacts of seasonality are greater for higher worm burdens.

## 5 Discussion

This paper has considered the effects of seasonality on the dynamics of *Ascaris* infection, and its implications for effective control of the infection using repeated drug treatment.

A mathematical model for the four stages of the life-cycle has been used to determine the influence of seasonal parameters on the mechanisms of the transmission process. Seasonal parameters influencing the dynamics of transmission are related to the development and survival properties of eggs and larvae in the environment, which are known to be affected by variations in temperature and moisture levels. In many settings where *Ascaris* infection is prevalent these are precisely the conditions which can vary significantly over an annual cycle. Mathematical techniques have been used to obtain an analytical solution for the stable oscillatory solution (i.e. the state before treatment) for a test case where seasonal parameters have an assumed annual sinusoidal variation. This determines explicit analytical expressions for the time lags between peaks in temperature and eggs, and through the subsequent life-cycle stages to mature worms. The solution presented here is for a simple generic set of functional forms for the key seasonal parameters. However it should be stressed that the technique is applicable to more complex annual profiles, since any profile can be described as a sum of any number of such simple forms. When the effects of drug treatment are investigated it is found that the optimal treatment time in the year coincides with the time when eggs and larvae are at a minimum and hence when reinfection is lowest. In terms of a more measurable quantity this coincides, for the scenario considered here, with when the temperature is near maximum. The analytical result can be used to interpret this optimal timing in terms of quantities that can be directly measured and could be a useful tool for predicting the most appropriate time to administer treatment.

The results obtained are shown to be robust and repeatable. Different assumptions for egg numbers and mean worm burden distributions (such as the negative binomial and random distributions) have been considered and the conclusions are found to be corroborated.

An important observation to note is that in isolation the impact of seasonality on the mean worm burden might appear to be insignificant. Indeed in the test case scenario investigated here the influence of seasonal changes only brought about a swing of  ± 2 in the variation in mean worm burden across the year compared to the equilibrium value in the absence of seasonality. However, when multiple treatments are considered the effect is much more significant. Treatment and seasonality disrupt the transmission cycle, and minima in egg and larvae numbers start to coincide with the minimum in mean worm numbers which amplifies the seasonal impact and the effectiveness of drug treatment. This leads to clearly defined optimal treatment times where significant reductions in mean worm burden can be achieved with fewer drug treatments compared to treating at other times of the year, or to possible local elimination if treating at the optimal time.

The model used in this paper has assumed exponential stages for the uninfected eggs and juvenile worms in that there are single egg and juvenile worm classes, with some loss due to death at the end of the development periods for each stage. Perhaps a more realistic approach would be to have these stages represented by an Erlang (integer gamma) distribution. In this case it is assumed that there are multiple egg and juvenile worm classes within the overall development periods, and loss due to death at the end of each of these subclasses. This model has been described and considered in the Supplementary Material. Inclusion of 4 egg and 4 juvenile worm stages, with multiple annual treatments, is found to produce results which are qualitatively similar to those for the exponential stages shown in the main paper. There is still a large window of optimum treatment times, which falls within the optimum treatment range predicted with the assumption of exponential stages in the main paper. This is an important result in terms of treatment program development and shows that the assumption of exponential stages is able to capture the significant qualitative effects of seasonality.

In some previous cases a selective drug treatment strategy has been proposed, where treatment is repeatedly focussed on individuals who have a predisposition to high worm burdens. The idea behind this approach is that selective treatment of those most heavily infected can lead to a reduction in viable eggs in the environment. However it has been found that selective treatment was not sufficiently effective and certainly not as cost effective as targeted (children aged 2–15) or mass treatment strategies (Asaolu, Holland, Crompton, 1991, Holland, O’Shea, Asaolu, Turley, Crompton., 1996). The results shown here suggest that the effects of seasonal forcing are of greater significance when mean worm burdens are high, which could be a contributing factor in the lack of success of such a targeted approach.

The practicalities of administering drug treatments in the field based on seasonal influences also needs to be considered. The results presented here suggest that for the test case investigated there is about a 6 week window, around the annual peak in temperature, for the administration of drugs to be optimal. The question is whether a mass treatment program could be applied successfully in the field over this optimum period of time. Administration of vaccines in a pulsed vaccination campaign, where vaccines are administered over a fixed, short period of time, rather than throughout the year, has proved both effective and practical - in particular the use of synchronised national immunisation days as part of the strategy for the global eradication of polio (Aylward and Heymann, 2005).

This paper has concentrated on the effects of an assumed temperature variation, but the key observation here is that it is the seasonal variation of the parameters concerned with egg and larvae development and survival in the environment which is important. The conclusions could be applied equally to variations associated with rainfall, for example. This would be a more typical seasonal variation in tropical climates where temperature variation is less but rainfall variation is significant. In addition to these developmental parameters varying throughout the year, the uptake parameter, *β*, may also be seasonal. This could be either owing to more eggs being exposed in wet conditions or children playing outdoors more in the school holidays when they would be more likely to be in contact with eggs in the soil than at other times of the year. The results presented here suggest that these variations could also play a role in the transmission cycle.

The purpose of this paper was to determine whether seasonal variations may have an influence on the dynamics of *Ascaris* infection. It should be mentioned that other helminths and parasites have free-living stages that are even more environmentally sensitive than *Ascaris*. For example seasonal variation in temperature can induce significant changes to the life-expectancies of the free-living infective larvae of hookworm, or the miracidia or cercaria of schistosomes (Anderson and May, 1991), so the methods developed in this paper may be even more significant in these other systems. The results presented here, and those of another theoretical study (Davis et al., 2018), strongly suggest that seasonal variation may have significant consequences for the effectiveness of any control program. The results certainly suggest that further experimental, field and mathematical study is necessary in order to fully understand the dynamics and control in cases where seasonal variation is evident.

## 6 Funding

AJC and TDH acknowledge funding from EPSRC Institutional Sponsorship of the University of Warwick from the Global Challenges Research Fund (EP/R512916/1). They also gratefully acknowledge funding of the NTD Modelling Consortium by the Bill and Melinda Gates Foundation (OPP1053230) in partnership with the Task Force for Global Health. The views, opinions, assumptions or any other information set out in this article should not be attributed to Bill & Melinda Gates Foundation and The Task Force for Global Health or any person connected with them.

## Appendix A Analytical solution for the stable oscillatory state

Suppose

$$\begin{array}{ccc} E(t) & = & E^{*}+ɛ\hat{E}e^{i\omega t}\\ L(t) & = & L^{*}+ɛ\hat{L}e^{i\omega t}\\ J(t) & = & J^{*}+ɛ\hat{J}e^{i\omega t}\\ M(t) & = & M^{*}+ɛ\hat{M}e^{i\omega t} \end{array}$$

where [*E**, *L**, *J**, *M**] are the equilibrium values established in the absence of seasonality, $\left[\hat{E},\hat{L},\hat{J},\hat{M}\right]$ are the amplitudes resulting from seasonality, and ε is the (small) seasonality parameter.

The egg production term *λ*(*M*)*M* can be expanded in powers of ε. As a first approximation only the leading-order term, $\lambda_{0}zM^{*}/{\left(1+M^{*}\left(1-z\right)/k\right)}^{k+1}=\lambda^{*},$ is used in the subsequent analysis, since it can be argued that the density dependent form of *λ*(*M*)*M* serves to establish an equilibrium, and that higher-order terms are small, especially if the mean worm burden, *M**, is large. For this analysis the mating function is set to unity.

### A1 Assuming only *τ_E_* is seasonal

Substitution into the equation for the variation in eggs gives

$$\frac{dE^{*}}{dt}+ɛi\omega \hat{E}e^{i\omega t}=\sigma N\lambda^{*}-\frac{1}{\tau_{E}^{*}}\left(1-ɛ\hat{\tau_{E}}e^{i\omega t}\right)\left(E^{*}+ɛ\hat{E}e^{i\omega t}\right).$$

At leading order the equilibrium value is determined:

(A.1) $$E^{*}=\sigma N\lambda^{*}\tau_{E}^{*}.$$

Equating *O*(ε) terms gives the solution for the amplitude correction:

(A.2) $$\hat{E}=\frac{{\hat{\tau }}_{E}}{\sqrt{1+\omega^{2}\tau_{E}^{*2}}}e^{-i\theta }E^{*},$$

where $\theta =\tan^{-1}\left(\omega \tau_{E}^{*}\right)$.

The equation for larvae, at leading order, is:

$$\frac{dL^{*}}{dt}=\frac{s_{E}}{\tau_{E}^{*}}E^{*}-\beta_{L}L^{*},$$

where $\beta_{L}=\beta N+\mu_{L}^{*},$ so that the equilibrium value is

(A.3) $$L^{*}=\frac{s_{E}}{\tau_{E}^{*}\beta_{L}}E^{*}.$$

The *O*(ε) equation for the larvae is:

$$i\omega \hat{L}=\frac{s_{E}}{\tau_{E}^{*}}\left(\hat{E}-{\hat{\tau }}_{E}E^{*}\right)-\beta_{L}\hat{L},$$

so that

(A.4) $$\hat{L}=\frac{e^{-\frac{i\pi }{2}}\omega s_{E}}{\sqrt{\omega^{2}+\beta_{L}^{2}}}e^{-i\psi }\hat{E}=\frac{e^{-\frac{i\pi }{2}}\omega \tau_{E}^{*}\beta_{L}}{\sqrt{\omega^{2}+\beta_{L}^{2}}}\frac{{\hat{\tau }}_{E}}{\sqrt{1+\omega^{2}\tau_{E}^{*2}}}e^{-i\psi -i\theta }L^{*},$$

where $\psi =\tan^{-1}\left(\omega /\beta_{L}\right)$.

The equation for juvenile worms, at leading order, is:

$$\frac{dJ^{*}}{dt}=\beta L^{*}-\xi_{J}J^{*},$$

where $\xi_{J}=1/\tau_{J}+\mu ,$ so that the equilibrium value is

(A.5) $$J^{*}=\frac{\beta }{\xi_{J}}L^{*}.$$

At *O*(ε):

$$i\omega \hat{J}=\beta \hat{L}-\left(\frac{1}{\tau_{J}}+\mu \right)\hat{J},$$

so that

(A.6) $$\begin{array}{ccc} \hat{J} & = & \frac{\beta }{\sqrt{\omega^{2}+\xi_{J}^{2}}}e^{-i\alpha }\hat{L}\\ & = & \frac{\xi_{J}}{\sqrt{\omega^{2}+\xi_{J}^{2}}}\frac{e^{-\frac{i\pi }{2}}\omega \tau_{E}^{*}\beta_{L}}{\sqrt{\omega^{2}+\beta_{L}^{2}}}\frac{{\hat{\tau }}_{E}}{\sqrt{1+\omega^{2}\tau_{E}^{*2}}}e^{-i\alpha -i\psi -i\theta }J^{*}, \end{array}$$

where $\alpha =\tan^{-1}\left(\omega /\xi_{J}\right)$.

Finally the equation for mature worms, at leading order, is:

$$\frac{dM^{*}}{dt}=\frac{s_{J}}{\tau_{J}}J^{*}-\mu_{M}M^{*},$$

so that the equilibrium value is

(A.7) $$M^{*}=\frac{s_{J}}{\tau_{J}\mu_{M}}J^{*}.$$

At *O*(ε):

$$i\omega \hat{M}=\frac{s_{J}}{\tau_{J}}\hat{J}-\mu_{M}M^{*},$$

so that

(A.8) $$\begin{array}{ccc} \hat{M} & = & \frac{s_{J}}{\tau_{J}\sqrt{\omega^{2}+\mu_{M}^{2}}}e^{-i\gamma }\hat{J}\\ & = & \frac{\mu_{M}}{\sqrt{\omega^{2}+\mu_{M}^{2}}}\frac{\xi_{J}}{\sqrt{\omega^{2}+\xi_{J}^{2}}}\frac{e^{-\frac{i\pi }{2}}\omega \tau_{E}^{*}\beta_{L}}{\sqrt{\omega^{2}+\beta_{L}^{2}}}\\ & & \times \;\frac{{\hat{\tau }}_{E}}{\sqrt{1+\omega^{2}\tau_{E}^{*2}}}e^{-i\gamma -i\alpha -i\psi -i\theta }M^{*}, \end{array}$$

where $\gamma =\tan^{-1}\left(\omega /\mu_{M}\right)$.

### A2 Assuming only *μ_L_* is seasonal

In this case the equilibrium values remain the same, but the amplitudes of the *O*(ε) terms are now

(A.9) $$\begin{array}{ccc} \hat{E} & = & 0, \end{array}$$

(A.10) $$\begin{array}{ccc} \hat{L} & = & \frac{\mu_{L}^{*}{\hat{\mu }}_{L}}{\sqrt{\omega^{2}+\beta_{L}^{2}}}e^{-i\psi }L^{*}, \end{array}$$

(A.11) $$\begin{array}{ccc} \hat{J} & = & \frac{\xi_{J}}{\sqrt{\omega^{2}+\xi_{J}^{2}}}\frac{\mu_{L}^{*}{\hat{\mu }}_{L}}{\sqrt{\omega^{2}+\beta_{L}^{2}}}e^{-i\alpha -i\psi }J^{*}, \end{array}$$

(A.12) $$\begin{array}{ccc} \hat{M} & = & \frac{\mu_{M}}{\sqrt{\omega^{2}+\mu_{M}^{2}}}\frac{\xi_{J}}{\sqrt{\omega^{2}+\xi_{J}^{2}}}\frac{\mu_{L}^{*}{\hat{\mu }}_{L}}{\sqrt{\omega^{2}+\beta_{L}^{2}}}e^{-i\gamma -i\alpha -i\psi }M^{*}, \end{array}$$

where all parameters are defined as above.

### A3 Assuming only *s_E_* is seasonal

The *O*(ε) terms in this case are

(A.13) $$\begin{array}{ccc} \hat{E} & = & 0, \end{array}$$

(A.14) $$\begin{array}{ccc} \hat{L} & = & \frac{\beta_{L}{\hat{s}}_{E}}{\sqrt{\omega^{2}+\beta_{L}^{2}}}e^{-i\psi }L^{*}, \end{array}$$

(A.15) $$\begin{array}{ccc} \hat{J} & = & \frac{\xi_{J}}{\sqrt{\omega^{2}+\xi_{J}^{2}}}\frac{\beta_{L}{\hat{s}}_{E}}{\sqrt{\omega^{2}+\beta_{L}^{2}}}e^{-i\alpha -i\psi }J^{*}, \end{array}$$

(A.16) $$\begin{array}{ccc} \hat{M} & = & \frac{\mu_{M}}{\sqrt{\omega^{2}+\mu_{M}^{2}}}\frac{\xi_{J}}{\sqrt{\omega^{2}+\xi_{J}^{2}}}\frac{\beta_{L}{\hat{s}}_{E}}{\sqrt{\omega^{2}+\beta_{L}^{2}}}e^{-i\gamma -i\alpha -i\psi }M^{*}. \end{array}$$

## Appendix B Supplementary materials

Supplementary Data S1 Supplementary Raw Research Data. This is open data under the CC BY license http://creativecommons.org/licenses/by/4.0/ Supplementary Data S1
