# Supplementary material for: The impact of seasonality on the dynamics and control of Ascaris lumbricoides infections
Source: J Theor Biol. 2018 Sep 14;453:96–107. doi: 10.1016/j.jtbi.2018.05.025 (PMC6013298; doi:10.1016/j.jtbi.2018.05.025)
Supplement: Supplementary Data S1 — Supplementary Raw Research Data. This is open data under the CC BY license http://creativecommons.org/licenses/by/4.0/ [file mmc1.pdf]

The impact of seasonality on the dynamics and control of *Ascaris lumbricoides* infections - supplementary material.

A.J. Cooper[1], T.Déirdre Hollingsworth[1,2]

1 Zeeman Institute for Systems Biology and Infectious Disease Epidemiology Research, University of Warwick, Coventry, CV4 7AL, UK
2 Big Data Institute, Li Ka Shing Centre for Health Information and Discovery, University of Oxford, Oxford, OX1 2JD, UK

Results for a random distribution of worms.

The results in Figures S1-S5 are for a random distribution and correspond to Figures 2, 4 and 5-7 in the main paper which are for an assumed negative binomial distribution.

Here the egg production term is taken to be of the form:

$$\lambda(M) = \lambda_0(1 - e^{-zM})/M,$$

with $z = 0.6$. If a random distribution of parasites is assumed, and the worms are polygamous, then the mating function, given by Anderson & May [1], is

$$\phi(M) = 1 - e^{-M/2}.$$

References

1. Anderson RM, May RM. 1991 Infectious diseases of humans: dynamics and control. Oxford University Press, Oxford. ISBN: 9780198540403

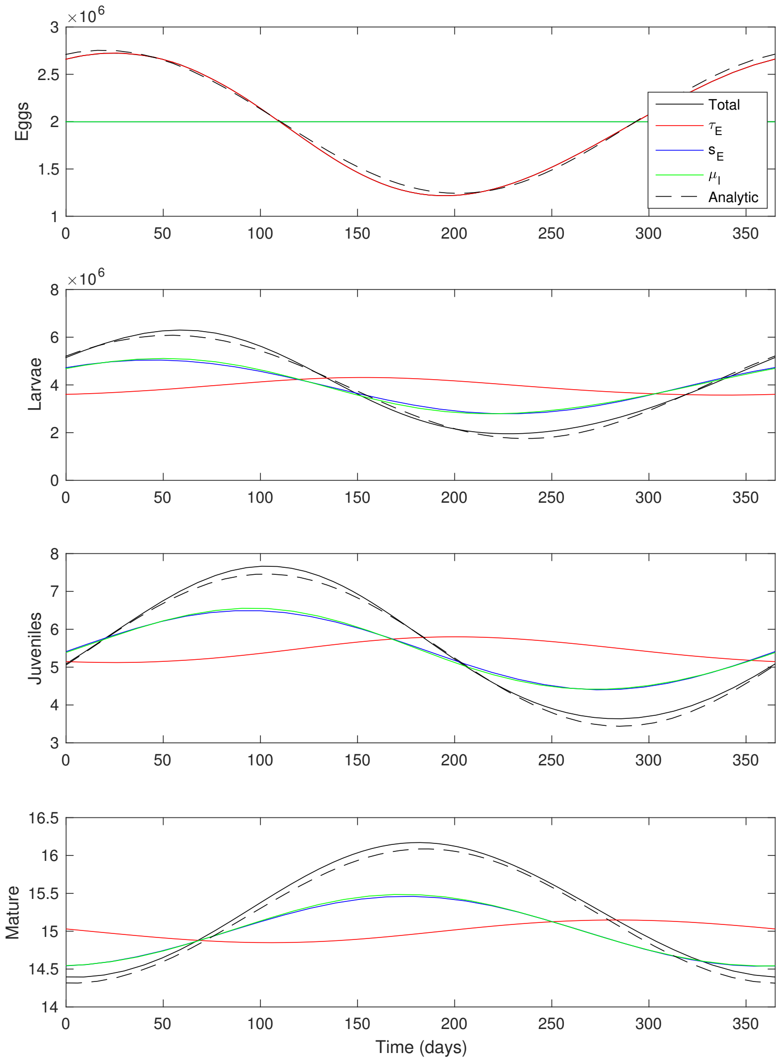

Figure S1: Effect of the different seasonal parameters τ_E^*, s_E^* and μ_L^* on the steady oscillatory solution, and comparison between the total numerical and analytical solutions. ($\varepsilon = 0.4$, $M^* = 15$)

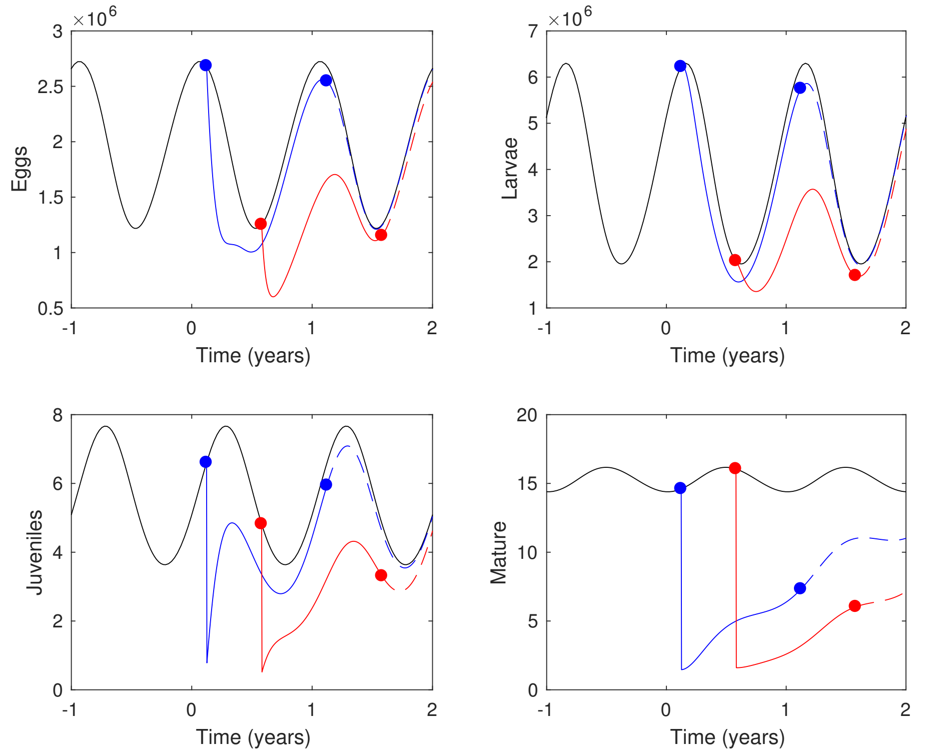

Figure S2: Effects of a single treatment. Time scale starts from zero in treatment year. Cases plotted include most effective (red) and least effective (blue) times to treat in terms of recovery of mature worms. Black lines indicate untreated case. Dots indicate time of treatment and time one year after treatment. Dashed lines indicate population levels if no further treatment occurs. Treatment efficacy is taken to be 90%.

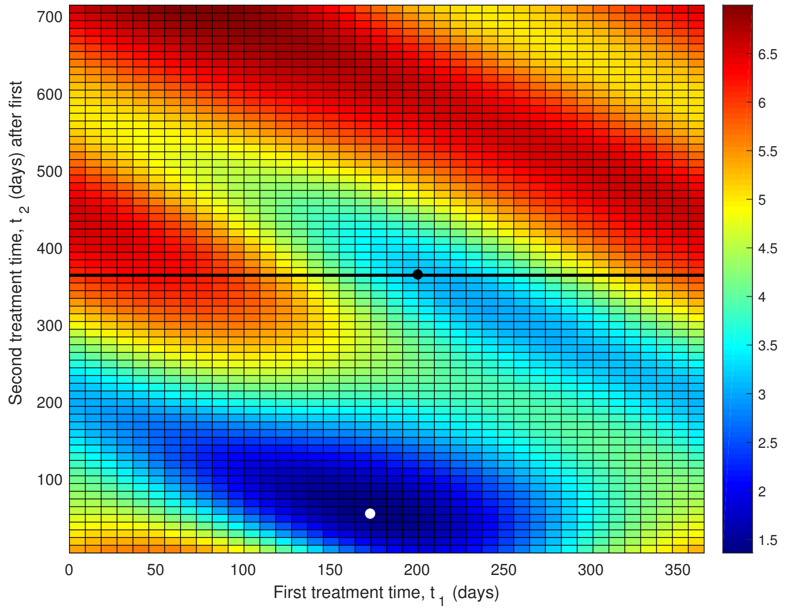

Figure S3: Timing of second treatment - value of M a year after second treatment. Treatment effectiveness = 90%, ε = 0.4, M^* = 15. White dot shows absolute minimum in M a year after 2 treatments. Black line shows second treatment a year after the first, with the minimum location along this line indicated by the black dot.

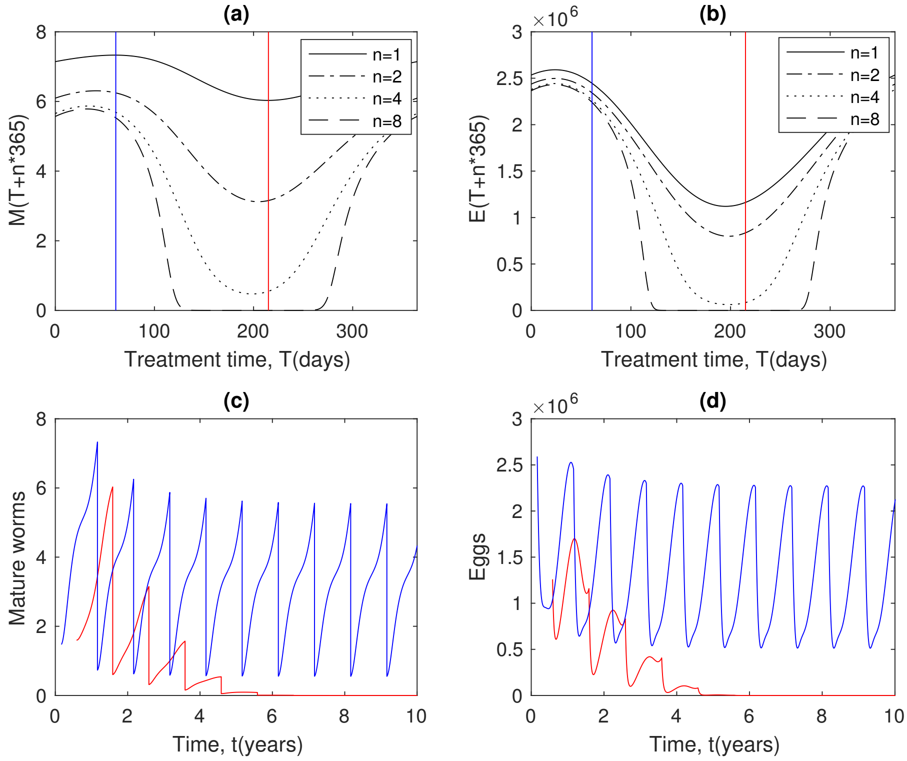

Figure S4: Effect of multiple annual treatments. (a) Variation in mean worm burden n years after initial treatment, as a function of treatment time, T. (b) Corresponding variation in egg numbers with treatment time. Red line indicates most effective time to treat ($T \approx 215$ days), blue line indicates least effective treatment time ($T \approx 60$ days). (c) Variation in mean worm burden in actual time, t, for most effective treatment time (red) and least effective treatment time (blue). (d) Variation in egg numbers throughout the treatment cycle at most effective (red) and least effective (blue) treatment times.

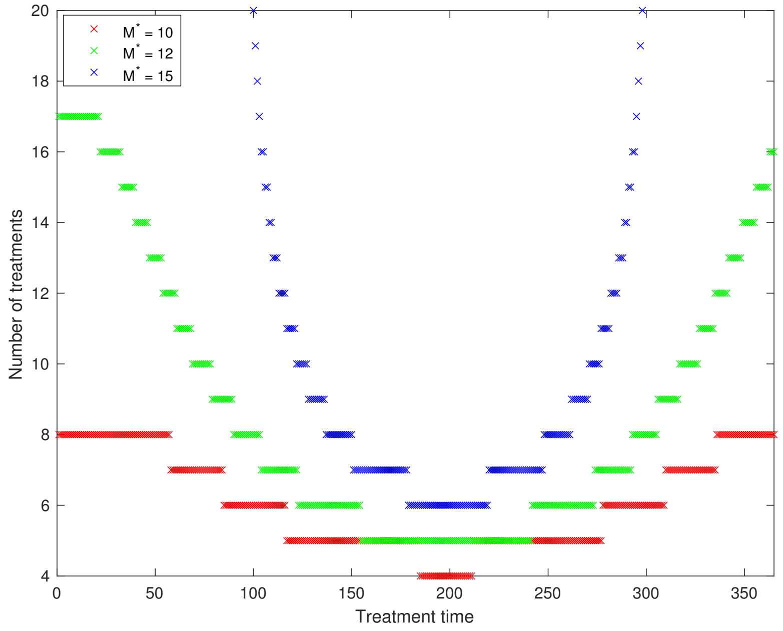

Figure S5: Number of treatments leading to local eradication for different equilibrium values of mean worm burden ($\varepsilon = 0.4$).

Multiple egg and juvenile worm classes.

The standard model assumes exponential stages for uninfected eggs and juvenile worms in that there are single egg and juvenile worm classes with some loss due to death (given by s_E and s_J) at the end of the development periods, τ_E and τ_J. Perhaps a more realistic approach is to have these stages represented by an Erlang distribution. In this case it is assumed that there are multiple egg and juvenile worm classes within the overall development periods, and loss due to death at the end of each of these subclasses. The model for this scenario is described below.

Consider the case where the egg class is divided into a number of subclasses. For example, if there are now k egg subclasses, and the maturation time for each egg class is taken to be equal, then the maturation time, $\tilde{\tau}_E$, associated with each subclass must satisfy:

$$\tilde{\tau}_E = \frac{\tau_E}{k}. \tag{1}$$

For the proportion of eggs surviving to the next subclass, $\tilde{s}_E$, the following relation must also hold:

$$\tilde{s}_E = \sqrt[k]{s_E}. \tag{2}$$

Similarly, for m juvenile worm subclasses, the maturation time and survival proportion for each stage is taken to be

$$\tilde{\tau}_J = \frac{\tau_J}{m}, \qquad \tilde{s}_J = \sqrt[m]{s_J}. \tag{3}$$

The governing equations for the complete life cycle then take the form:

$$\frac{dE_1}{dt} = \sigma N \phi(M)\lambda(M)M - \frac{E_1}{\tilde{\tau}_E}, \tag{4}$$

$$\frac{dE_i}{dt} = \frac{\tilde{s}_E E_{i-1}}{\tilde{\tau}_E} - \frac{E_i}{\tilde{\tau}_E}, \qquad i = 2, \dots k \tag{5}$$

$$\frac{dL}{dt} = \frac{\tilde{s}_E E_k}{\tilde{\tau}_E} - \beta_L L, \tag{6}$$

$$\frac{dJ_1}{dt} = \beta L - \left(\frac{1}{\tilde{\tau}_J} + \mu_H\right) J_1, \tag{7}$$

$$\frac{dJ_i}{dt} = \frac{\tilde{s}_J J_{i-1}}{\tilde{\tau}_J} - \left(\frac{1}{\tilde{\tau}_J} + \mu_H\right) J_i, \qquad i = 2, \dots m \tag{8}$$

$$\frac{dM}{dt} = \frac{\tilde{s}_J J_m}{\tilde{\tau}_J} - (\mu_M + \mu_H)M. \tag{9}$$

The effects of multiple annual treatments when there are for 4 egg and 4 juvenile worm stages are shown in Figure S6, which plots the mean worm burden against treatment time after n treatments. This shows that after multiple treatments the results are qualitatively similar to those for the standard model (as given by Figure 6 in the main paper). There is still a large window of optimum treatment times, which falls within the optimum treatment range predicted under the assumption of exponential stages in the main paper. This is an important result in terms of treatment program development. Also evident is that seasonal effects appear enhanced under the assumption of multiple egg and juvenile worm classes, in that outside of the optimum treatment times, treatments are even less effective compared to the optimum timing.

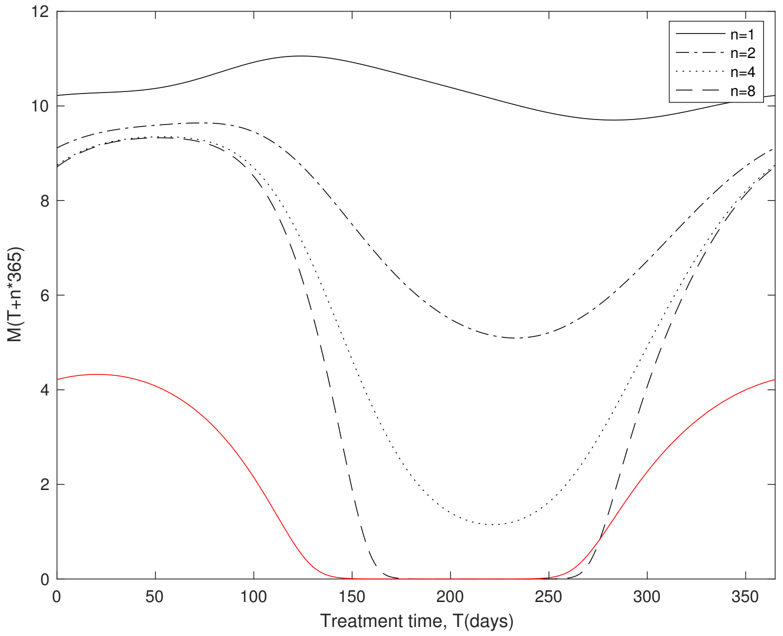

Figure S6: Effect of multiple egg and juvenile worm classes - Erlang distribution with 4 egg and 4 juvenile worm stages. $\varepsilon = 0.4$). Red line indicates the $n = 8$ case under the assumption of exponential stages for uninfected eggs and juvenile worms.
